# Enhancing Adolescent and Young Adult Health Services! A Review of the Community Needs Assessment Process in an Urban Federally Qualified Health Center

**DOI:** 10.1089/heq.2019.0108

**Published:** 2020-05-18

**Authors:** Jade C. Burns, Sierra Teadt, Wayne W. Bradley, George H. Shade

**Affiliations:** ^1^ School of Nursing, University of Michigan, Ann Arbor, Michigan, USA.; ^2^ School of Public Health, University of Michigan, Ann Arbor, Michigan, USA.; ^3^ Detroit Community Health Connection, Detroit, Michigan, USA.

**Keywords:** community needs assessment, federally qualified health center, adolescents, African American, service delivery

## Abstract

**Background:** Urban African American adolescents and young adults (AYAs) face multiple barriers to effective engagement in health care, including access to primary and specialty care services resulting in significant disparities in care.

**Purpose:** To conduct a needs assessment to enhance service delivery of AYAs at an urban federally qualified health center (FQHC) organization in Detroit.

**Methods:** Semistructured interviews were conducted among pediatric staff members (*N*=11) using the community needs assessment approach specified for FQHCs.

**Results:** The needs assessment determined the following priorities for AYAs seeking care at this FQHC organization: (1) mental health (e.g., depression and anxiety), (2) obesity, and (3) sexual health (e.g., sexually transmitted infection testing).

**Conclusion:** When analyzing a population to learn about community-based issues, a needs assessment is a valuable tool. The information here has been used as supplemental information to address the health inequities that African American youth face within Detroit.

## Introduction

Urban African American adolescents and young adults (AYAs) face multiple barriers to effective engagement in health care, resulting in significant disparities in care. For example, sexually transmitted infection (STI) rates are disproportionately high among African American AYAs aged 15–24 years.^1,2^ They also face many barriers to reproductive health care and health education, including inadequate STI screening by health care providers, lack of transportation, and limited access to insurance coverage.^3–6^ The purpose of this brief is to define high-priority issues of an AYA community, describe the community needs assessment (CNA) process, determine the resources available to address these problems, and enhance service delivery among this population at an urban school linked federally qualified health center (FQHC).

A CNA is a useful tool for identifying the strengths and resources a community has for addressing the social and health care needs of its members and must be culturally sensitive.^7,8^ FQHCs must perform a CNA every 3 years to accurately document the needs of the communities they serve and to receive federal designation funding for this purpose.^9,10^ According to Health Resources and Service Administration (HRSA) guidelines, these health centers may also focus on underserved subsets of their overall populations (e.g., children, elderly people, and people with HIV/AIDS); as well as their mission that is to improve the health status of marginalized communities and advocate for high-quality care.^8,9,11^

## Methods

The information gathered here was a part of a more extensive organization-wide CNA comprising seven FQHC ambulatory health centers in the city of Detroit, serving medically underserved populations. From the larger CNA, a total of 42 employees were interviewed by medical specialty as well as 460 patient satisfaction surveys were included to look at the population's health priorities, preferences regarding care, and the vital role that FQHCs play within the community. The interview guide was developed from the HRSA Compliance Manual^9^ and the University of Kansas Community Tool Box.^10^ The questions were reviewed by the executive leadership and partnering faculty for content to ensure that they met HRSA requirements. A semistructured interview method was used. Open-ended questions were asked, with 19 items covering the history of the organization and community, the health care services offered, the leading health problems, the unique issues faced by adolescents, resources, assets, and the sustainability of the community. The voluntary interviews lasted ∼60 to 90 min. Responses were manually coded, then summarized for thematic analysis. The themes from the interviews were used to describe AYA-prioritized health issues, provider-perceived health issues of the AYAs community, resources needed at the clinic, utilization, barriers, and social determinants of health from a staff member perspective. IRB review was not required as the assessment was a requirement by HRSA and did not include any interaction with human subjects or access to identifiable private information.

## Results

Eleven health care providers and staff members were interviewed. A summary of the predominant themes and notable responses from the staff are highlighted in Table 1.

**Table 1.** Results from the Community Needs Assessment Interviews

Questions	Health care providers/staff (n=11)
Description of Detroit Community Health Connection	
Series of federally qualified health centers in seven locations in metro area	3
Predominantly African American population	3
Low cost health care to insured and uninsured	3
Primary care, pediatrics, OBGYN, internal medicine, dental, WIC	3
Patients and employees from a range of communities (e.g., Hispanic, African American, Middle Eastern, Caucasian)	2
Provide care for communities with many needs (social, mental, physical)	2
Nonprofit	1
Predominately Hispanic and African American population	1
School-linked health center	1
Located in underserved areas	1
Serve Medicaid patients	1
Role at clinic	
Medical assistant	3
Physician	3
Pediatrician	2
Director	2
Nurse practitioner	1
Services offered at clinic	
Physicals	4
Sexual health (e.g., STI screening, pregnancy testing, birth control)	3
Dental	2
Immunizations	2
Internal medicine/adult medicine	1
Pediatrics	1
Behavioral health counseling	1
Transient social service	1
Weight checks	1
Blood work	1
Sign up for WIC services	1
Providing treatment plans that are standard of care and agreed upon by patient	1
Perceived health-related issues that adolescents prioritize	
Sexual health issues (e.g., STIs, pregnancies)	5
Mental and behavioral health issues (e.g., depression, anxiety, substance abuse)	1
Immunizations	1
Physicals	1
Skin problems (e.g., acne)	1
Asthma	1
Obesity	1
Colds/viruses	1
Pain	1
Clinic's significant assets and how they could be strengthened	
Should have stationary social workers rather than rotating them	2
Nutrition counseling	1
Refer patients, but need to ensure they can get to referral location	1
Service both physical and mental health	1
Access to pediatrician when clinic is open, 24 h access for urgent and immediate care	1
Should have more mental health resources and onsite mental health workers	1
Should have a stable workforce with an onsite center director	1
Should have more community outreach to ensure care that is needed is provided	1
Should train staff in other resources (e.g., social service, connection to community services)	1
Perceived main health problems for all patients coming to the clinic	
Mental and behavioral health issues (e.g., depression, anxiety, substance abuse)	6
Obesity	4
Lead exposure	3
Asthma/allergies	3
STIs	2
Hypertension	2
Substance abuse	1
Diabetes	1
Arthritis	1
Communicable diseases	1
Genetic disorders	1
Cerebral palsy	1
Autism	1
Failure to thrive	1
Poor nutrition	1
Abnormal lipid disorders	1
Lack of resources	1
Dental care	1
Top three perceived health problems of adolescents	
Mental and behavioral health issues (e.g., depression, anxiety, substance abuse)	8
Obesity	6
Sexual health issues (e.g., STIs, pregnancies)	5
Asthma/allergies	2
Communicable diseases	1
Health issues specifically affecting adolescents and young adults	
Mental health and behavioral health issues (e.g., depression, anxiety, substance abuse)	2
Sexual health issues	1
Homelessness, transient home environments	1
Access to food	1
Daily living resources	1
Lack of transportation	1
Individuals affected disproportionately by health issues	
Entire population, does not matter which ethnic group you are from	1
Families affected after manufacturing jobs left	1
African Americans	1
LGBTQ+ community	1
Low income	1
Risk factors that contribute to health issues in adolescents and young adults	
Built environment (e.g., absence of jobs, failing schools, lack of resources)	3
Lack of education	2
Unprotected sex	2
Poverty	1
Lack of funds	1
Lack of transportation	1
Television/music	1
Food choices	1
Peer pressure, bullying, violence	1
How many adolescents are affected by health issues	
Sexual health issues: 70–75%	1
Mental and behavioral health issues: 8–11%	1
Obesity: 50%	1
Diabetes, hypertension, or arthritis: 70%	1
Frequency of health issues arising	
Two pregnancies per week	1
One to two STIs per week	1
Mental and behavioral health issues increasing rapidly	1
See patients with asthma daily	1
More frequently over time, as more people lose wages within the community	1
More than usual	1
Most important health issues of adolescents	
Sexual health issues (e.g., STIs)	2
Mental and behavioral health	2
Obesity	1
Barriers and challenges to addressing health issues	
Teens feeling invincible/prioritization of health/not accepting responsibility	4
Lack of transportation	3
Lack of insurance	2
Lack of education	1
Behavioral service referrals are a cumbersome process	1
How health issues could be reduced and/or eliminated	
Tailored education and health promotion	4
Transportation	2
Work with community outreach to get name out there	1
Attack problem proactively	1
More support on hand	1
Youth programs	1
Resources currently available at the clinic to address health issues	
Referral system	5
Expertise of providers	4
Social workers	2
Brown bags (free condoms)	2
Brochures/information from CDC	2
Prescriptions	1
Nutrition services (registered dietician, nutrition counselor)	1
Fit kids program	1
Resources needed to better address health issues	
More material information/education for teens	4
Transportation	2
Social services on site	2
Updated esthetics	1
More exposures and marketing	1
More resources for uninsured and underinsured	1
Ample staff on site with ample training	1
Scheduling follow-ups	1
What is needed at the clinic to sustain the clinic's strengths	
Funding	1
Transportation	1
More resources	1
Staff	1
Birth control (e.g., condoms)	1
$1 million question	
Larger space/updated esthetics	4
Youth programs (e.g., reading/math, cooking, exercise)	3
Transportation	2
Condoms/contraceptives	2
More exposure and marketing	2
Ongoing training/continued education for existing staff	2
Full-time dietician	1
More education (e.g., proper diet, rest, medications, mental health)	1
More money on confidential services and behavioral health services	1
Pediatric urgent care	1
Help with scheduling visits	1
Diagnostic testing for those who cannot afford it	1
Additional comments	
Funding for prepaid laboratory forms and medication	1
Esthetics of facility	1
Education	1
Transportation	1
Counseling	1
Better insurance coverage	1

CDC, Centers for Disease Control and Prevention; LGBTQ+, lesbian, gay, bisexual, transgender and queer/questioning +, OBGYN, obstetrics and gynecology; STI, sexually transmitted infection; WIC, women, infants, and children.

### Top three perceived health care problems of adolescents

**FIG. 1.**
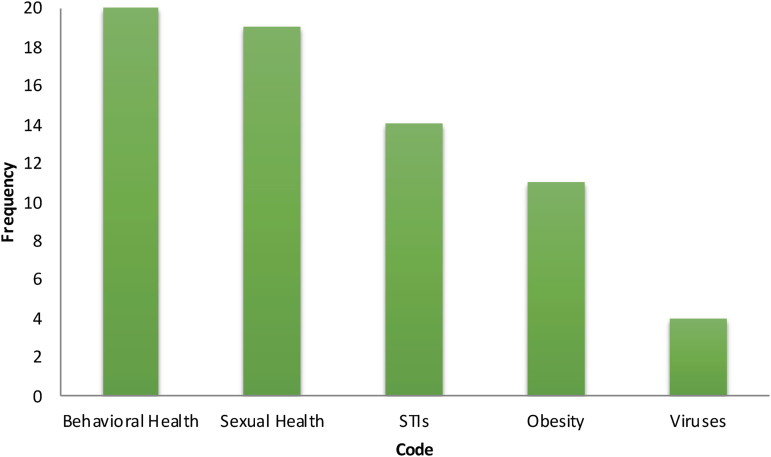
Adolescents and young adults prioritized health issues. STI, sexually transmitted infections.

The three most prioritized health issues included mental and behavioral health, obesity, and sexual health (see Fig. 1). These problems were reported to be the most prevalent in African American, low-income, and sexual minority (lesbian, gay, bisexual, transgender, and queer/questioning) AYA populations. The number of AYA males who obtained these services was also disproportionate in comparison with AYA females. Approximately 70–75% of adolescents were stated affected by sexual health issues, and more than half are affected by obesity or related chronic conditions such as hypertension.

Behavioral health screenings were a priority for providers. Anxiety and depression were diagnosed frequently in AYA patients, which is said to be due to a number of social determinants. Conversations about mental and behavioral health between patients and providers were usually initiated after results are reviewed from screening tools such as the Patient Health Questionnaire (PHQ)-2, PHQ-9, and rapid assessment for adolescent preventive services survey.

Marijuana and vaping were indicated to be one of the most frequently used drugs among patients. It was noted that marijuana was having a more significant impact on this population than anticipated. It was also reported through community forums, and discussions with patients, family members, teachers, and coaches, that are securing employment had been affected by positive screening results. Reduced performance on school examinations, difficulty maintaining attention, and delayed problem-solving abilities were also evident. The interviews also indicated that increased risk-taking, poor judgment, and consent problems arose with marijuana use.

### Risk factors for health problems in AYAs

Risk factors found from the interviews in this community, poor public transit systems, safety, and assistance having prescriptions filled at local pharmacies minutes away from the clinic, are among the social determinants of quality of care for this population. The assessment also indicated that anxiety and depression are heightened in adolescents who live in poverty or are economically disadvantaged. AYAs who live in shelters and are consistently unsure when they will have money or food undoubtedly experience higher levels of stress. The interviews indicated that living in the urban environment of downtown Detroit was also not conducive to AYA health and contributed to risky behavior. One individual stated “adolescents are faced with the absence of jobs, failing schools, peer pressure, bullying, and gang-related violence, among other factors.” AYAs who must deal with anxiety, stress, and depression “often try to escape these feelings through engaging in risky behaviors like unsafe sex, unhealthy eating, and marijuana use.”

The CNA indicated that the lack of fresh food is a risk factor for obesity in this community. There are no grocery stores for miles around other than convenience stores that carry mainly snack foods and a supermarket chain store that specializes in organic food. The store above has been centrally located since 2013 and does offer a variety of healthy food choices; most people in the community cannot afford to shop there.

### Staff perceptions

Providing holistic care, focusing on interpersonal and behavioral health of adolescents, and collaborating with individuals and organizations that are equally engaged and dedicated to community health were important to staff members. Solutions included additional staff training, partnering with Lyft or Uber to improve attendance at appointments; reinitiating the youth advisory council for feedback and advice; sharing case studies in pediatric provider meetings; interviewing the AYA population to tailor programs to them; and developing educational and health promotional materials to improve the health of this population.

## Discussion

The CNA process provided an opportunity to be exposed to interprofessional teams and service-learning experience to undergraduate and graduate students in public health and nursing. Through conducting semistructured interviews, students were able to (1) understand the role structure and function of, and the population served by the health center and (2) evaluate experiences and approaches to working with diverse communities.^12^ It also uncovered risk factors, health problems, and unhealthy behaviors among the AYA population that were similar to the current AYA health literature such as depression, homelessness, and nutrition and weight issues.^1,13–15^ A strength of the assessment is that the organization that this school-linked clinic provides services with extended hours and intensive care for sexual, reproductive, and fertility health, and behavior health, such as substance-abuse planning. Also, AYAs have access to care at this center regardless of their ability to pay. The center has a program that is funded to cover these costs, with a broad range of sliding scale health services, of which most patients utilize.

Our CNA does have limitations that include (1) small sample size, (2) perspectives from only providers and staff; AYAs themselves were not interviewed, and the results might have been different if AYA perspectives and opinions were incorporated, and (3) these results are not be generalizable to other populations.^14^ CNAs are context specific and valid only for a given population, location, and time, so the results of this assessment may not be applicable to other populations.^14^ To remedy these limitations, larger sample should be interviewed in the future. In addition, AYAs in the community should be interviewed to ensure that their perceptions of the community's needs align with those of the health care providers and staff members.

## Conclusion

FQHCs play a significant role in improved geographic access in delivering comprehensive primary health services to individuals of all ages in underserved settings.^16^ This assessment, along with future research, will serve as a guideline for the development and implementation of interventions aimed at promoting the use of community health centers and reducing gaps in care, specifically among young people in Detroit. The creation of this interview guide has helped to highlight a specialized population with unique needs, identify barriers that match the literature, as well as strengths and available resources within the organization and community: a process that may be replicated by staff within this larger FQHC organization. The results of this CNA have been disseminated to community members and stakeholders. Funding and IRB approval have been received to look at the data through a mixed-methods lens, specifically at young men's reproductive health called the Stay Safe Project.

## Abbreviations Used

AYAs: adolescents and young adults
CDC: Centers for Disease Control and Prevention
CNA: community needs assessment
FQHC: federally qualified health center
HRSA: Health Resources and Service Administration
STI: sexually transmitted infection
WIC: women, infants, and children
